# Ophthalmia Neonatorum

**Published:** 1910-03-12

**Authors:** Sydney Stephenson

**Affiliations:** Ophthalmic Surgeon to Queen Charlotte's Hospital, London, etc.


### Hospital Clinics.

### OPHTHALMIA NEONATORUM.

By SYDNEY STEPHENSON, F.R.C.S., Ophthalmic Surgeon to Queen Charlotte's Hospital, London, etc.

(A Lecture delivered at the Polyclinic, London, W.C., on January 10, 1910.)

Gentlemen,—I make no apology for selecting so trite a subject as Ophthalmia Neonatorum for this lecture. The disease in question is the cause of at least two-thirds of all the blindness in the world, and is, moreover, preventable. These facts have been recognised for many years, and yet the ravages of the malady go unchecked. In other words, a preventable disease is not prevented.

Now almost all labours in this country are attended either by medical men or midwives. Although it is impossible to get at the exact figures, yet it appears that the cases are about equally divided between the two classes. Ophthalmia does not seem to be more frequent among midwives than among medical men, a somewhat disconcerting reflection. I find that of 62 cases of ophthalmia neonatorum seen by me in hospital practice a medical man had been in attendance upon the mother in 37—that is, in more than one half. In America the facts appear to be substantially the same. Dr. W. O. Moore[1] found that of 500 cases of ophthalmia treated in a period of ten years at the New York Eye and Ear Infirmary only 50 had been delivered by midwives. The others had been under the charge of practitioners of medicine. Again, Dr. W. A. Francisco,[2] in a list of 40 cases of ophthalmia neonatorum found that labour had been conducted in 19 instances by medical practitioners and in 21 by midwives. Dr. F. Park Lewis, chairman of the committee on ophthalmia neonatorum of the American Medical Association, has recently stated: "So long as half the births are attended by partially-trained midwives and many more by not over-careful physicians, one thing is necessary: the widest dissemination of information as to the importance, the danger, and the prevalence of infantile ophthalmia."[3] Finally, to return to British figures, Mr. Stephen Mayou[4] investigated 162 confinements complicated with ophthalmia, and ascertained that 90 had been attended by medical men and 72 by midwives.

### A Preventable Calamity.

It cannot be repeated too often that 99 out of every 100 cases of ophthalmia neonatorum can be avoided if proper precautions are taken at birth. Its occurrence, in my opinion, reflects no credit upon whomsoever, be it doctor or midwife, is in charge of the labour. It implies, as a rule, a degree of carelessness that is likely to add little to the reputation of those immediately concerned. I am convinced that until this point is fully appreciated no great progress will be made in the prevention of the disease. The sooner that we as a profession recognise this fact the better, since that portion of the general public interested in blindness and its prevention is alive to the facts, and before very long will demand an explanation of every case of ophthalmia which occurs in the practice of either medical man or midwife. It behoves us, therefore, thoroughly to understand the pathogeny of the disease, and, above all, its prevention.

Indeed, during the last few years the medical profession has shown commendable activity in all that concerns ophthalmia neonatorum. In 1903 a discussion upon its prevention took place at the Obstetrical Society of London. The British Medical Association appointed a committee on ophthalmia neonatorum, the report of which was published in the *Journal* of May 8th last. The American Medical Association also has had a committee at active work for some little time.

The Central Midwives Board, under the control of which all women who practise midwifery for gain will come from and after the first day of April next, have for long scheduled ophthalmia as a condition in which medical help must be sent for by the midwife. Within the last few months the Board has gone farther, and has included the presence of purulent discharge from the pregnant or parturient woman as a condition in which the midwife must summon medical help. The Board has also recently issued an excellent leaflet dealing with inflammation of the eyes in newborn children, and directing that preventive measures should be taken in all cases of birth. Finally, the borough of Stoke-upon-Trent has placed ophthalmia upon the list of diseases which are compulsorily notifiable to the Public Health Authorities. Yet the malady is not prevented, nor will it ever be until such time as we can bring home to everybody concerned the responsibility as regards the baby's eyes that devolves upon all who conduct a labour case.

### Definition and Diagnosis.

What is ophthalmia neonatorum? It is an inflammatory disease affecting the eyes of babies within a few days of birth, and characterised by redness and swelling of the eyelids, together with the escape of purulent discharge from the eyes. But there is ophthalmia and ophthalmia. Some cases are extremely mild and speedily recover with or without treatment. Others, on the contrary, are so severe that in the absence of skilled treatment sight may be impaired or lost altogether. It is essential to distinguish clearly between these two forms. Fortunately, the distinction is not usually difficult. To begin with, there are the clinical characters of the disease—mild in the one case and severe in the other. The sovereign test, however, lies in the bacteriological examination of the discharge that escapes from between the baby's eyelids. Generally speaking, severe cases of ophthalmia neonatorum are characterised by the presence of the gonococcus in the secretion, whereas the others show various other micro-organisms, such as the pneumococcus, the bacterium coli, the Koch-Weeks' bacillus, and ordinary pyococci, to name some of the more important only. It must not be assumed that gonococci can always be found in discharge from the inflamed eyes. Among 1,493 cases of ophthalmia neonatorum gonococci could be found in 68.13 per cent., or about two-thirds of the cases. Every case of ophthalmia, therefore, is not of venereal origin, and does not imply a lapse from morality on the part of one or other parent.

In round numbers, it may be stated that of every hundred cases of ophthalmia neonatorum 65 per cent. are associated with gonococci, 10 per cent. with pneumococci, 5 per cent. with bacterium coli, 5 per cent. with other pathogenic micro-organisms, and, finally, 15 per cent. with negative bacteriological findings not to be accounted for on the ground of incomplete bacteriological examination (Axenfeld). Most of the last-named cases are due, I believe, to congenital syphilis.

Therefore, when called to a baby with ophthalmia the first thing is to prepare films from the discharge and either to examine them for yourselves or else get somebody else to do so. If gonococci be found, the greatest circumspection must be exercised in the treatment of the case, no matter how apparently slight may be the symptoms. Remember that it is such cases that blind the babies.

### Types of the Disease.

We distinguish two types of infantile ophthalmia, primary and secondary, according to the time that has elapsed between birth and the appearance of symptoms. Primary ophthalmia declares itself within five days after labour, while secondary ophthalmia comes on at some later time. The distinction is not without practical importance. Primary cases are always due to inoculation of the baby's eyes with infective material at, or, more commonly, immediately after birth. *Prima facie* it might be thought that infection would be most likely to occur while the baby's head was passing through the maternal passages, and such a view, I believe, is still current in certain text-books. Vaginal infection, however, is not common for two reasons. First, that the junction between the eyelids is for all practical purposes water-tight; and secondly, that it is often thickly coated with vernix caseosa. It was reserved for an English ophthalmic surgeon, the late Mr. P. H. Mules, of Manchester, to point out a much more likely method of infection, namely, by direct action of the perineal edge. His idea was that during the passage of the fœtal head through the external orifice the anterior edge of the perineum, at that moment a tight, elastic, curved cord, pressed upon the baby's eyelids and deposited infective secretion between them.[5]

At the same time it is now very generally recognised that infection usually reaches the infant's eyes soon after the head is born, by such material gaining access to the conjunctival sac either by the repeated blinking of the baby or by the baby infecting his own eyes by rubbing them with infected knuckles. Another very important way whereby disease may be conveyed to the eye is by the use of the same water in the first bath for both face and body.

### Ætiology.

The mechanism of so-called secondary ophthalmia is not always quite so clear. But it usually results from the conveyance of infective lochia to the eyes either by the fondlings of the mother, the carelessness of the midwife, or the fingers of the child. In extremely rare cases the nurse herself may be the *fons et origo mali*, since, upon examination, she may be found to be affected with gonorrhœa. Apart from these the working rule is that every case of primary or secondary ophthalmia results from the transfer (direct or indirect) of infective secretion from the mother to the child.

A word or two should be said about what is sometimes called ante-partum ophthalmia, a rare but by no means an unknown condition. By the expression we mean either that a baby is born with symptoms of ophthalmia or else that he develops the disease within a few hours of birth. In these cases it is clear that infection must have taken place before the child actually entered the world. It is commonly taught that such cases are met with only when the second stage of labour has been unduly prolonged. It is believed that after rupture of the membranes and escape of the "waters" gonococci or other micro-organisms have been enabled to reach the conjunctival sac, had time to develop *in situ*, and thus to produce the clinical appearances of ophthalmia. This view is not quite correct. In 71 cases of ante-partum ophthalmia recently collected by me the explanation could apply in 26 only, thereby leaving 45 cases to be accounted for in some other way. I believe myself that most cases of ante-partum ophthalmia, not due to contamination of the eyes after premature escape of the waters, can be best explained on the theory of a local intra-uterine infection. The recent experiments of Hellendahl[6] appear to establish the fact that such is possible.

These cases present an obvious analogy to those rare cases where the liquor amnii is offensive to the sense of smell, sometimes horribly so. Under these circumstances the bacterium coli has been found in the fluid. That organism, it has been surmised, may have ascended from the mother's anus, have descended from the Fallopian tube, or may even have passed through the uterine wall from the neighbouring intestine (M. Handfield Jones).

### Some Fallacies.

There are one or two fallacies concerning ophthalmia neonatorum that may be mentioned:

1. It is sometimes assumed that the mother of a baby with ophthalmia must be affected with acute gonorrhœa. Nothing could well be farther from the truth. As we have already seen, in only about two-thirds of the cases can gonococci be found in the infant's eyes. The truth of the matter is that acute ophthalmia in the baby usually results from chronic gonorrhœa in the mother. Of twenty women with affected babies, Groenouw[7] observed pain during micturition in 15 per cent. only, despite the fact that all the women suffered from vaginal discharge containing gonococci. A history of profuse yellow discharge and scalding was obtained in 17 per cent. only of my own cases. The underlying infective condition is much more commonly a latent gonorrhœa, producing an endocervicitis, metritis, or urethritis, and perhaps occasioning no discomfort at all to the woman. Many of the women, I am convinced in my own mind, are quite ignorant that they are infected in any way. This point is of importance, and accounts for the disconcerting way in which cases of ophthalmia may crop up in the most unexpected quarters. Cases are known of ophthalmia in a baby long years after infection in the father. Thus, Gayet has reported a case of a baby which developed ophthalmia on the second day.[8] Gonococci were found (1) in discharge from the baby's eye and (2) in the mother's vaginal secretion on the eleventh day after confinement. The father admitted gonorrhœa ten years before, and gonococci were discovered in the "military drop" from which he had suffered ever since. Other cases of the sort, including some personal ones, might be quoted.

2. Another fallacy is that cases of ophthalmia coming under treatment before the cornea is involved can be cured without damage to sight. This fallacy has led to actions at law against medical men for alleged negligence. Schanz has recently reported such a case from Germany. The truth of the matter is that a clear cornea justifies a relatively but not an absolutely good prognosis. I have myself seen eyes go wrong under the best conditions as regards treatment. In May, 1888, I showed a baby at the Polyclinic which had lost both eyes, although the mother was delivered in Queen Charlotte's Hospital, and the eyes had been under my own care almost from the first.

3. A third fallacy is that cases due to the gonococcus are always severe, and that they can be diagnosed by mere clinical inspection. It is true that most cases of gono-ophthalmia can be identified as such after a little experience. It is also true that, as a rule, such cases last for several weeks, which is not generally so when the conjunctival inflammation is due to organisms other than the gonococcus. At the same time the only scientific plan is to demonstrate the gonococci. There are mild—so-called "catarrhal"—cases that can be identified as gonorrhœal by no amount of clinical acumen. It is not easy to state the proportion of such cases, but Dr. J. E. Weeks, of New York, in six cases where gonococci were present, had two only of a severe description.[9] "Abortive" cases also are not unknown, where symptoms, never very marked, disappear in a surprisingly short time under almost any kind of treatment. The importance of these "catarrhal" and "abortive" cases lies in the fact that, despite their apparent mildness, they may at almost any moment be complicated with corneal mischief, as in cases observed by Groenouw, Druais, and myself.

4. The last fallacy that I shall mention is the belief that ophthalmia neonatorum is invariably a local affection. On the contrary, in rare instances the disease may become systemic and give rise to arthritis, pleuritis, peritonitis, endocarditis, meningitis, septicæmia, and pyæmia. Death has resulted more than once (Widmark, Chartres, Stevens, Lewis, etc.). It is of interest to recall the fact that the swollen joints that occasionally accompany ophthalmia neonatorum were first described by an English surgeon, Mr. R. Clement Lucas.[10] A few months later a Swedish ophthalmologist, Johan Widmark, described such cases independently.[11]

### Prophylactic Treatment.

From very early times attempts of a primitive nature have been made to prevent ophthalmia neonatorum. But to an Englishman, Dr. Benjamin Gibson, of Manchester, who wrote in the year 1807, we owe the first clearly reasoned description of the rational means of preventing the disease. Here is what he said in a communication that will for ever be memorable in the annals of ophthalmia neonatorum: "First, to remove, if possible, the disease in the mother during pregnancy; second, if that cannot be accomplished, to remove artificially as much of the discharge as possible from the vagina at the time of delivery; and third, to pay at all events particular attention to the eyes of the child by washing them immediately after delivery with a liquid calculated to remove the offending matter or to prevent its noxious action."[12]

It is of some slight historical interest to note that in 1837 it was proposed in the Spanish Cortes to enjoin that infants should be baptised in warm water, since it was believed that destructive ophthalmia resulted from the chill incurred in the performance of that Christian rite.

But the year 1881 marked an era in preventive medicine, for Professor Credé, of Leipsig, then published details of his famous method, the application of a single drop of a 2-per-cent. solution of silver nitrate into the baby's eyes as soon as convenient after birth.[13] The success of Credé's method has been fully established wherever it has been adopted. In the year 1907 I collected figures dealing with 76,452 infants treated with the silver solution, and the percentage of ophthalmia among them amounted to 0.703 only. At the same time we must look facts in the face and acknowledge that there are certain drawbacks to anything like the general adoption of the plan. In the first place, the putting of the silver into the eyes is followed in almost every instance by more or less reaction, as shown by discharge and, less commonly, by redness. Secondly, quite a number of cases have been reported where the use of the Credé method was succeeded by persistent or even fatal bleeding from the conjunctiva. These were doubtless examples of the so-called "hæmorrhagic disease of the newly-born," and it is doubtful how far the silver was responsible for the bleeding. Thirdly, ulcerations or opacities of the cornea are said to have followed the use of the silver drops, although the statement must be accepted with much reserve. In short, we must admit that the Credé method is most efficient in preventing ophthalmia, and that with the exception of a more or less trivial catarrh of the conjunctiva it does no harm. In pre-antiseptic days, when the ravages of ophthalmia may be better imagined than described, we were compelled to put up with the drawbacks of the Credé plan, in view of its contingent advantages. But we now know that good results may be obtained from the employment of less irritating agents to the baby's eyes. Foremost among these stand out 1 per cent. solution of silver nitrate, 25 per cent. solution of argyrol, and 10 per cent. to 15 per cent. solution of protargol. Any one of these agents is most efficient in preventing ophthalmia.

I do not wish to convey the impression, however, that, in order to secure immunity from ophthalmia, a chemical agent must invariably be applied to the eyes. Hill (1805), Jacob (1834), Middlemore (1835), Hocken (1843), Whitehead (1847), Haynes Walton (1872), and other early nineteenth-century writers urged the necessity of cleansing the baby's eyes immediately after birth. In 1879 we find Dr. Samuel Hague, a general practitioner of Camberwell, advising that the eyes be wiped free from every trace of moisture the instant the head was born and before the baby had time to open his eyes. Hague quoted no figures, but he made the significant statement that he had attended to this point in hundreds of cases and had never known it to fail, whereas in the same period ophthalmia had repeatedly occurred in cases where the child had been born before his arrival.[14]

In fact, it has now been shown that removal of every trace of discharge from the baby's eyelids, if carried out as soon as the head is born, with pledgets of cotton wool or other clean material, will reduce ophthalmia well-nigh to the vanishing point. This is the procedure enjoined upon midwives by the leaflet recently issued under the authority of the Central Midwives Board. It is simple, harmless, and most efficient. No objection can possibly be raised to its adoption in every case of normal labour. In my opinion, the medical attendant would be well advised to assure himself that it is properly carried out in every instance, or even to do it himself if he entertains any doubt as to the competence of the midwife.

When gonorrhœa is known or suspected to exist further steps must be adopted, as by dropping into the baby's eyes one of the solutions mentioned before. I myself prefer the 1 per cent. solution of silver nitrate for the purpose. This, I consider, should be no part of the midwife's duty, but should always be carried out by a medical man, called in, if necessary, specially for the purpose.

You will thus see that we have at our disposal two methods of prevention, an *aseptic* and an *antiseptic* one. The former aims at preventing infective germs from reaching the baby's eye; the latter at destroying such when they have gained entrance. That both are efficient can be denied by nobody who is acquainted with the circumstances. The only weak point of the aseptic method is that it will assuredly fail should micro-organisms have entered during the actual progress of the labour, but that, as I have endeavoured to show, is not a common event.

In conclusion, let me urge that preventive measures be adopted in all cases, whether the mother suffers from a purulent vaginal discharge or not. I suggest that the aseptic plan will be efficient in cases presumably normal as regards danger of ophthalmia neonatorum, while in the others the antiseptic plan should be used in addition.

---

[1] *Medical Record*, September 30, 1879.
[2] *N.Y. Eye and Ear Infirmary Reports*, Jan. 1895.
[3] *New York State Journal of Medicine*, April 1907.
[4] *Practitioner*, 1908, p. 359.
[5] *Medical Chronicle*, January 1888.
[6] *Beit. zur Geb. u, Gynäk.*, Bd. X, Heft 2.
[7] V. Graefe's *Archiv f. Ophthalmologie*, Bd. LII., 1901.
[8] *Lyon Médical*, 1886, p. 350.
[9] *Medical Record*, July 24, 1886.
[10] *British Medical Journal*, February 1885.
[11] *Hygeia*, August 1885.
[12] *Edin. Med. and Surg. Journal*, 1807, p. 160.
[13] *Archiv f. Gynäk.*, 1881.
[14] *British Medical Journal*, June 21, 1879.

